# Supplementary material for: Immune System Deficiencies Do Not Alter SARS-CoV-2 Evolutionary Rate but Favour the Emergence of Mutations by Extending Viral Persistence
Source: Viruses. 2024 Mar 13;16(3):447. doi: 10.3390/v16030447 (PMC10974344; doi:10.3390/v16030447)
Supplement: Supplementary file 1 [file viruses-16-00447-s001.zip › viruses-2901742-supplementary.pdf]

Supplementary Material

Immune system deficiencies do not alter SARS-CoV-2 evolutionary rate but favour the emergence of mutations by extending viral persistence

Laura Manuto[1§], Martina Bado[1§], Marco Cola[1], Elena Vanzo[1], Maria Antonello[1], Giorgia Mazzotti[1], Monia Pacenti[2], Giampaolo Cordioli, Lolita Sasset[2], Anna Maria Cattelan[1,2], Stefano Toppo[1*], Enrico Lavezzo[1*]

[§]These authors contributed equally to this work

[*]Authors jointly supervised this work

[1]University of Padova, Padova, Italy

[2]University Hospital of Padova, Padova, Italy

Generation of consensus genomes

Paired-end sequences were first cleaned with Trimmometic (java -jar trimmomatic-0.39.jar PE -phred33 file_R1.fastq file_R2.fastq file_R1_paired file_R1_left file_R2_paired file_R2_left ILLUMINACLIP:TruSeq3-PE-2:2:30:10 LEADING:30 TRAILING:30 SLIDINGWINDOW:4:20 MINLEN:40), then aligned to SARS-CoV-2 reference genome with BWA-MEM (bwa mem -M -t 12 SARS-CoV-2_ref_genome.fasta file_R1_paired file_R2_paired >file_aligned.sam. Samtools was used to convert sam files into indexed and sorted bam files, which were then dereplicated with PICARD (java -jar picard.jar MarkDuplicates -I sorted_file.bam -M file_metrics --REMOVE_DUPLICATES true --REMOVE_SEQUENCING_DUPLICATES true --VALIDATION_STRINGENCY SILENT -O dereplicated_file.bam). Samtools was then utilised to generate mpileup files (samtools mpileup -f SARS-CoV-2_ref_genome.fasta dereplicated_file.bam -B -Q 0 -d 0 -a -o file.mpileup), necessary for variant calling with VarScan without imposing any filter (java -jar VarScan.v2.3.9.jar mpileup2cns file.mpileup --min-coverage 0 --min-var-freq 0 --output-vcf 1 >no_filter_file.vcf). Then, an in-house Python script selected reliable mutations applying more stringent filters to low coverage samples and taking into account of mutation frequency and support of forward and reverse reads. Eventually, the consensus genome was obtained with bcftools (cat SARS-CoV-2_ref_genome.fasta | bcftools-1.17/bcftools consensus -H A cons.vcf.gz -o consensus.fasta).

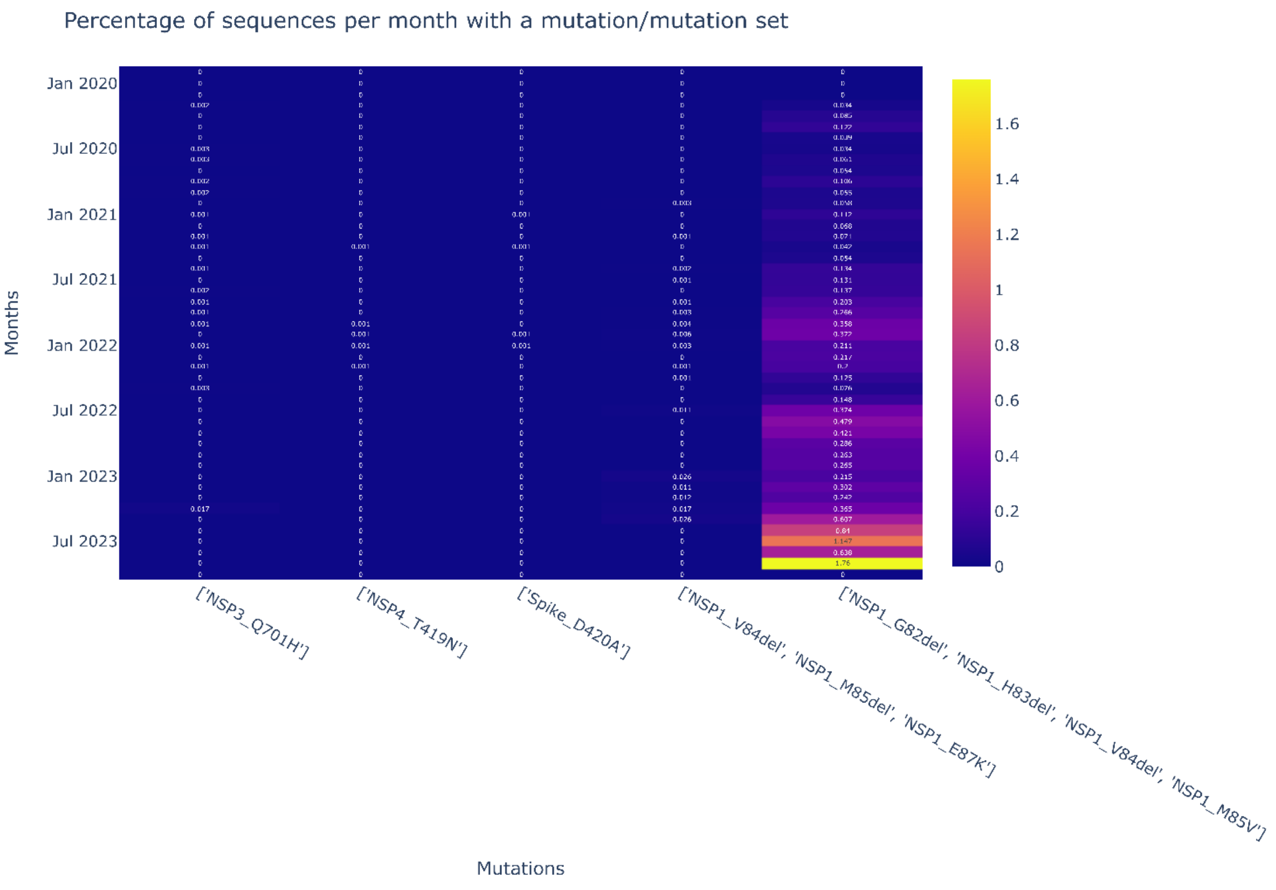

Figure S1. Frequency of the emerged non-synonymous mutations in GISAID. *The heatmap reports the monthly frequency of SARS-CoV-2 sequences available in GISAID and carrying either NSP3_Q701H, NSP4_T419N, S: D420A, 519_524del or 510_518del. Frequency is expressed as the number of sequences carrying the mutation of interest divided by the total number of available sequences for that month. Only complete sequences sampled in humans, with high coverage and with the date including year and month were considered, according to GISAID metadata.*

Date	*['NSP3_Q701H']*	*['NSP4_T419N']*	*['Spike_D420A']*	*['NSP1_V84del', 'NSP1_M85del', 'NSP1_E87K']*	*['NSP1_G82del', 'NSP1_H83del', 'NSP1_V84del', 'NSP1_M85V']*
2019-12	0	0	0	0	0
2020-01	0	0	0	0	0
2020-02	0	0	0	0	0
2020-03	0.002295263	0	0	0	0.034428939
2020-04	0	0	0	0	0.084959762
2020-05	0	0	0	0	0.12208908
2020-06	0	0	0	0	0.039408867
2020-07	0.002849896	0	0	0	0.034198752
2020-08	0.002777392	0	0	0	0.061102625
2020-09	0	0	0	0	0.053821313
2020-10	0.001565337	0	0	0	0.106442928
2020-11	0.002356129	0	0	0	0.055369029
2020-12	0	0	0	0.003297147	0.057700075
2021-01	0.001023468	0	0.001023468	0	0.11206976
2021-02	0	0	0	0	0.06817581
2021-03	0.000595043	0	0	0.000595043	0.07051263
2021-04	0.000591398	0.000591398	0.000591398	0	0.041989225
2021-05	0.000373343	0.000373343	0.000373343	0	0.053761434
2021-06	0.000925939	0	0	0.001851878	0.133798155
2021-07	0	0.00024031	0.00024031	0.00072093	0.130728692
2021-08	0.001840784	0	0	0.000460196	0.137445237
2021-09	0.000576844	0.000192281	0.000384563	0.000769126	0.202856918
2021-10	0.000611582	0	0.000407721	0.003057911	0.265630511
2021-11	0.001465729	0.000651435	0.000325718	0.004234328	0.357637831
2021-12	0.000479036	0.001437109	0.00119759	0.005987952	0.371732075
2022-01	0.001128044	0.001128044	0.001128044	0.003384133	0.210944286
2022-02	0	0	0	0	0.216584996
2022-03	0.00097996	0.00097996	0	0.00097996	0.199911804
2022-04	0	0	0	0.001083459	0.124597766
2022-05	0.00276606	0	0	0	0.076066662
2022-06	0	0	0	0	0.147973457
2022-07	0	0	0	0.011335298	0.374064838
2022-08	0	0	0	0	0.47897818
2022-09	0	0	0	0	0.420757363
2022-10	0	0	0	0	0.286289895
2022-11	0	0	0	0	0.263092987
2022-12	0	0	0	0	0.265404203
2023-01	0	0	0	0.025819778	0.215164816
2023-02	0	0	0	0.011190689	0.302148612
2023-03	0	0	0	0.012100678	0.242013553
2023-04	0.017391304	0	0	0.017391304	0.365217391
2023-05	0	0	0	0.026378264	0.606700079
2023-06	0	0	0	0	0.839895013
2023-07	0	0	0	0	1.147342995
2023-08	0	0	0	0	0.637522769
2023-09	0	0	0	0	1.759530792

2023-10	0	0	0	0	0

Table S1. *Monthly frequency of SARS-CoV-2 sequences available in GISAID and carrying either NSP3_Q701H, NSP4_T419N, S: D420A, 519_524del or 510_518del.*

a

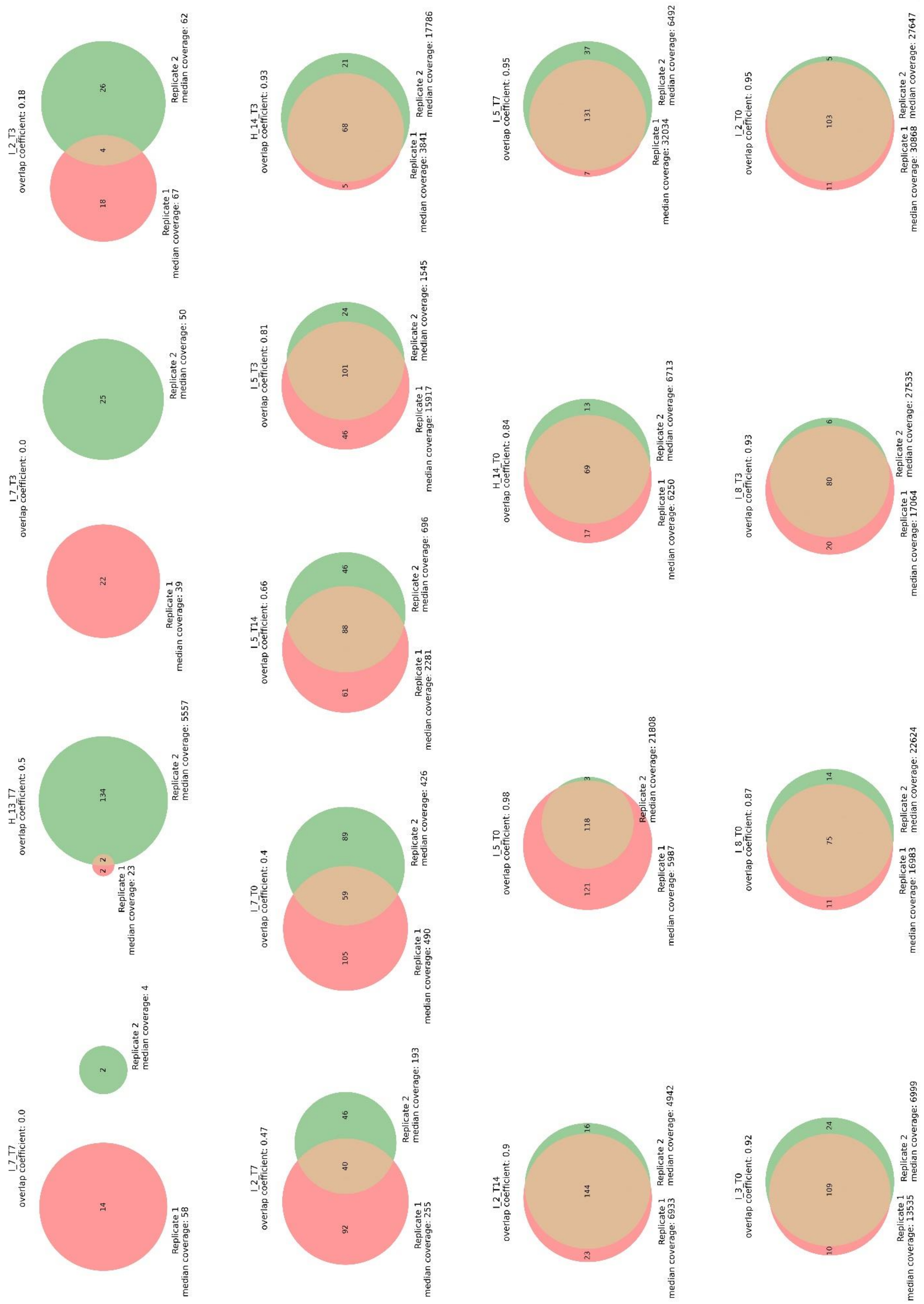

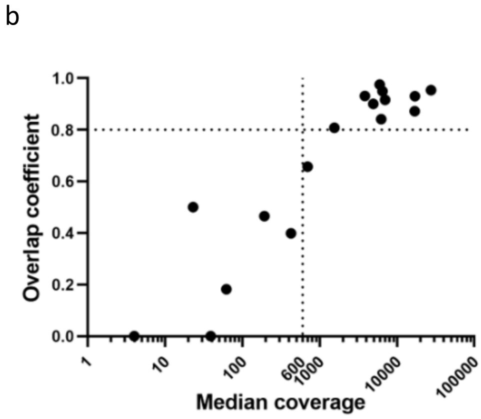

Figure S2. Minor variants reproducibility. *Overlap coefficient of minor variants identified in both replicates of the 17 samples that were sequenced twice. For a minor variant to be called, its frequency must be at least 1% and lower than 50%. a) Venn diagrams showing the overlap coefficient per each sample sequenced twice. The name of each sample is reported on top of Venn diagrams, while the median coverage of each replicate is provided below the relative circle. b) Overlap coefficient correlation with median coverage.*

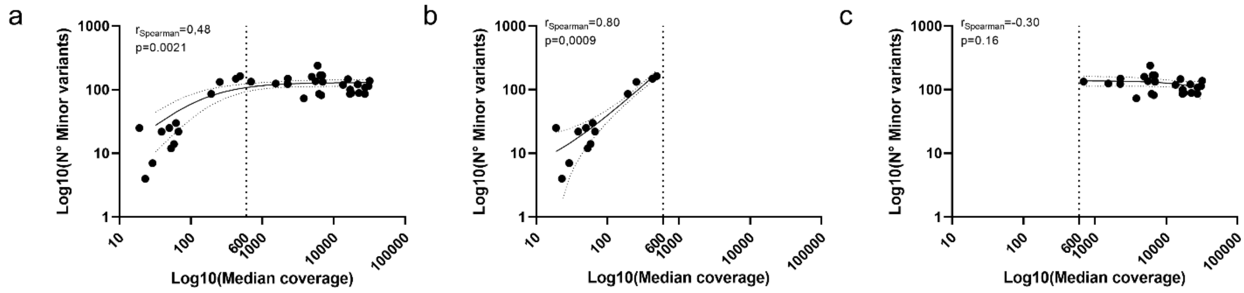

Figure S3. Correlation between minor variants and median coverage. *The correlation between the number of minor variants called and the sequencing depth of the sample was investigated on a) all the samples sequenced twice, b) on the samples with median coverage below 600 reads per genomic position, c) on the samples with median coverage equal or greater than 600 reads per genomic position. The Spearman correlation with the relative p value is provided for each scenario.*

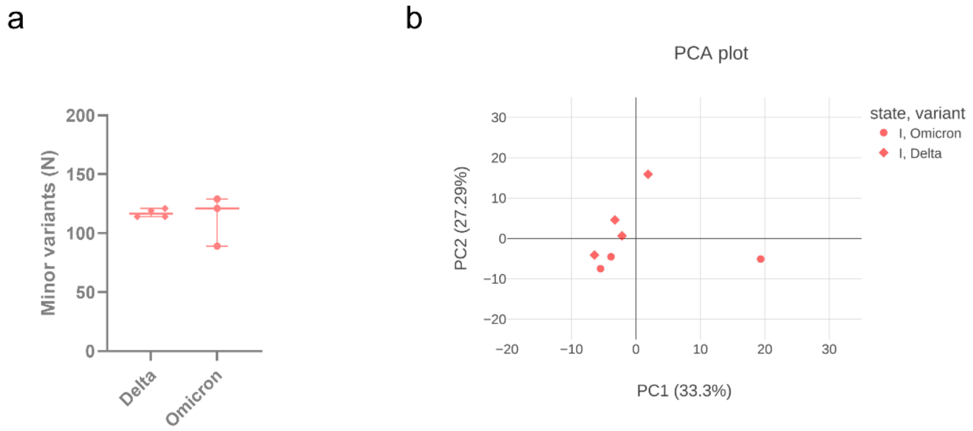

Figure S4. Delta and Omicron minor variants are comparable in number and type. *a) Number of minor variants identified at T0 in immunocompromised subjects that were infected with Omicron variant compared with the ones identified in subjects infected with Delta variant (N_{Delta}=4, $Median_{Delta}$=116.5, $N_{Omicron}$=3, $Median_{Omicron}$=121.0, p=0.8, Mann-Whitney test). b) Principal component analysis of the minor variants detected in Delta and Omicron samples. The variation explained by each PC is provided along the relative axis.*

GenBank_ID	GISAID ID	Immune state	Sample N°	T	Full_ID	CollectionDate	Consensus	MinVars
OR841441	EPI_ISL_18541893	H	1	T3	H_1_T3	27/12/2021	x	x
OR841443	EPI_ISL_18541894	H	1	T7	H_1_T7	31/12/2021	x	
OR841445	EPI_ISL_18541906	H	2	T0	H_2_T0	15/02/2022	x	x
OR841446	EPI_ISL_18541907	H	3	T0	H_3_T0	15/02/2022	x	x
OR841447	EPI_ISL_18541908	H	4	T0	H_4_T0	16/02/2022	x	x
OR841448	EPI_ISL_18541909	H	4	T2	H_4_T2	18/02/2022	x	
OR841453	EPI_ISL_18541910	H	5	T0	H_5_T0	15/03/2022	x	
OR841459	EPI_ISL_18541911	H	5	T7	H_5_T7	22/03/2022	x	
OR841452	EPI_ISL_18541912	H	6	T0	H_6_T0	15/03/2022	x	
OR841460	EPI_ISL_18541913	H	6	T7	H_6_T7	22/03/2022	x	
OR841454	EPI_ISL_18541914	H	7	T0	H_7_T0	16/03/2022	x	x
OR841455	EPI_ISL_18541915	H	7	T3	H_7_T3	18/03/2022	x	
OR841461	EPI_ISL_18541916	H	7	T7	H_7_T7	23/03/2022	x	
OR841456	EPI_ISL_18541917	H	9	T0	H_9_T0	17/03/2022	x	x
OR841462	EPI_ISL_18541918	H	9	T7	H_9_T7	23/03/2022	x	x
OR841457	EPI_ISL_18541895	H	10	T0	H_10_T0	16/03/2022	x	x
OR841458	EPI_ISL_18541896	H	11	T0	H_11_T0	21/03/2022	x	
OR841463	EPI_ISL_18541897	H	11	T7	H_11_T7	28/03/2022	x	x
OR841464	EPI_ISL_18541898	H	12	T0	H_12_T0	29/03/2022	x	x
OR841465	EPI_ISL_18541899	H	12	T3	H_12_T3	01/04/2022	x	
OR841434	EPI_ISL_18541900	H	13	T0	H_13_T0	15/02/2022	x	x
OR841450	EPI_ISL_18541901	H	13	T3	H_13_T3	18/02/2022	x	
OR841418	EPI_ISL_18541902	H	13	T7	H_13_T7	22/02/2022	x	x
OR841419	EPI_ISL_18541903	H	14	T0	H_14_T0	10/03/2022	x	x
OR841420	EPI_ISL_18541904	H	14	T3	H_14_T3	14/03/2022	x	x
OR841421	EPI_ISL_18541905	H	14	T7	H_14_T7	18/03/2022	x	
OR841435	EPI_ISL_18541919	I	1	T0	I_1_T0	14/12/2021	x	x
OR841436	EPI_ISL_18541920	I	1	T3	I_1_T3	17/12/2021	x	
OR841437	EPI_ISL_18541921	I	1	T7	I_1_T7	21/12/2021	x	
OR841423	EPI_ISL_18541922	I	2	T0	I_2_T0	20/12/2021	x	x
OR841415	EPI_ISL_18541924	I	2	T3	I_2_T3	23/12/2021	x	
OR841425	EPI_ISL_18541925	I	2	T7	I_2_T7	27/12/2021	x	
OR841428	EPI_ISL_18541923	I	2	T14	I_2_T14	03/01/2022	x	x
OR841424	EPI_ISL_18541926	I	3	T0	I_3_T0	22/12/2021	x	x
OR841439	EPI_ISL_18541927	I	3	T3	I_3_T3	25/12/2021	x	x
OR841426	EPI_ISL_18541928	I	3	T7	I_3_T7	29/12/2021	x	x
OR841438	EPI_ISL_18541929	I	4	T0	I_4_T0	22/12/2021	x	x
OR841440	EPI_ISL_18541930	I	4	T3	I_4_T3	25/12/2021	x	x
OR841442	EPI_ISL_18541931	I	4	T7	I_4_T7	29/12/2021	x	
OR841416	EPI_ISL_18541932	I	5	T0	I_5_T0	23/12/2021	x	x
OR841417	EPI_ISL_18541935	I	5	T3	I_5_T3	26/12/2021	x	x
OR841427	EPI_ISL_18541936	I	5	T7	I_5_T7	30/12/2021	x	x
OR841429	EPI_ISL_18541933	I	5	T14	I_5_T14	05/01/2022	x	x
OR841430	EPI_ISL_18541934	I	5	T21	I_5_T21	14/01/2022	x	
OR841444	EPI_ISL_18543347	I	6	T0	I_6_T0	14/02/2022	x	x
OR841449	EPI_ISL_18543348	I	6	T3	I_6_T3	18/02/2022	x	
OR841451	EPI_ISL_18543349	I	6	T7	I_6_T7	21/02/2022	x	x
OR841414	EPI_ISL_18541937	I	7	T0	I_7_T0	15/02/2022	x	
OR841431	EPI_ISL_18541938	I	7	T3	I_7_T3	18/02/2022	x	
OR841432	EPI_ISL_18541939	I	7	T7	I_7_T7	22/02/2022	x	

OR841422	EPI_ISL_18541940	I	8	T0	I_8_T0	29/03/2022	x	x
OR841433	EPI_ISL_18541941	I	8	T3	I_8_T3	01/04/2022	x	x

Table S2. *Full list of samples ID, collection data, consensus sequence availability and fulfilment of criteria for minor variants analysis.*

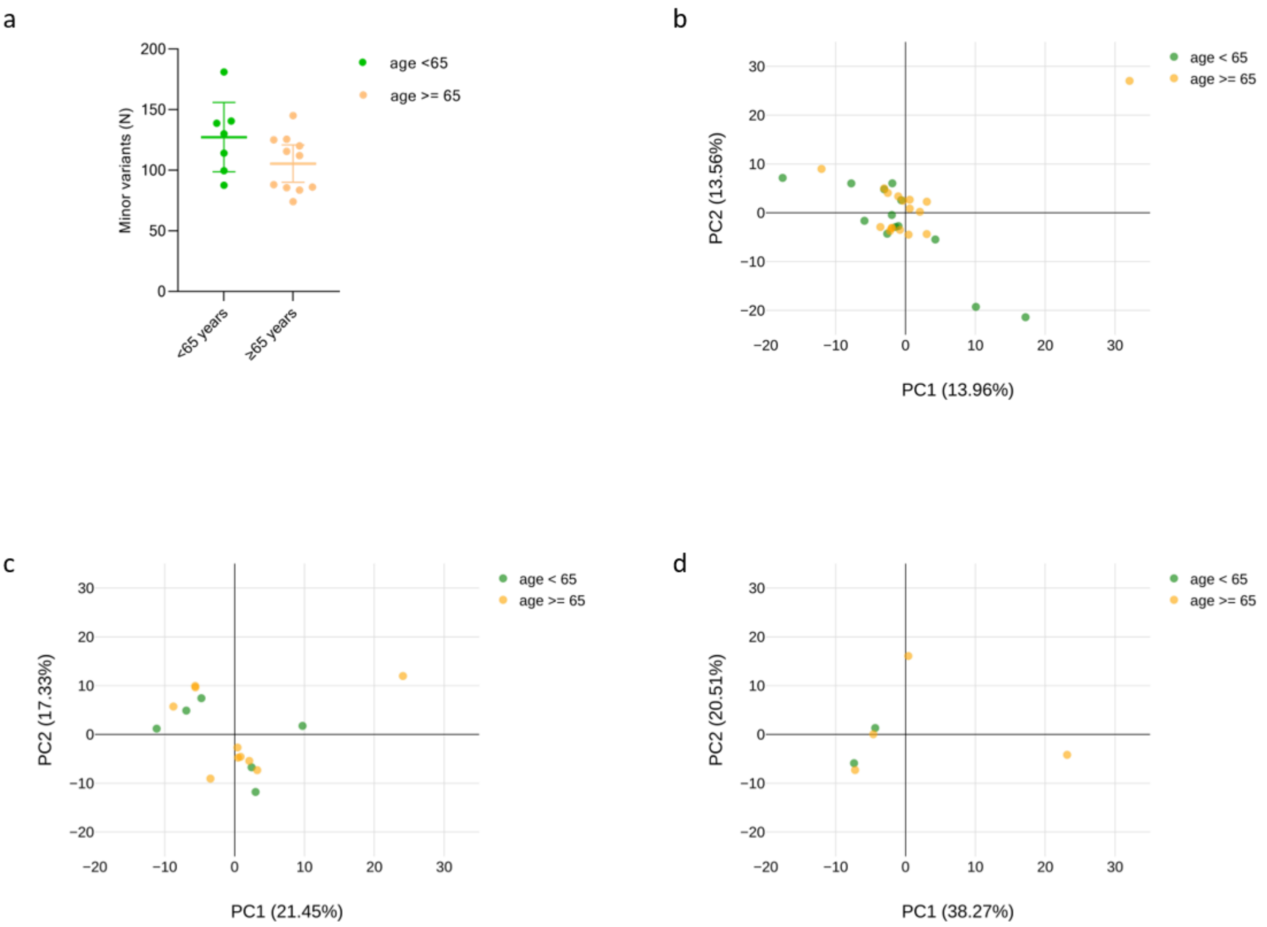

Figure S5. Age does not affect minor variants' number and type. *a) Comparison of the number of minor variants identified at any timepoint in subjects with less than 65 years, depicted in green, and older, reported in yellow (N<65=7, Mean<65 =127.3, N≥65=11, Mean≥65=105.5, p=0.10, unpaired t test). b) Principal component analysis comparing the minor variants detected at b) any timepoint, c) at T0 and d) at T7 in subjects less than 65 years old and older subjects. The variation explained by each PC is provided along the relative axis.*

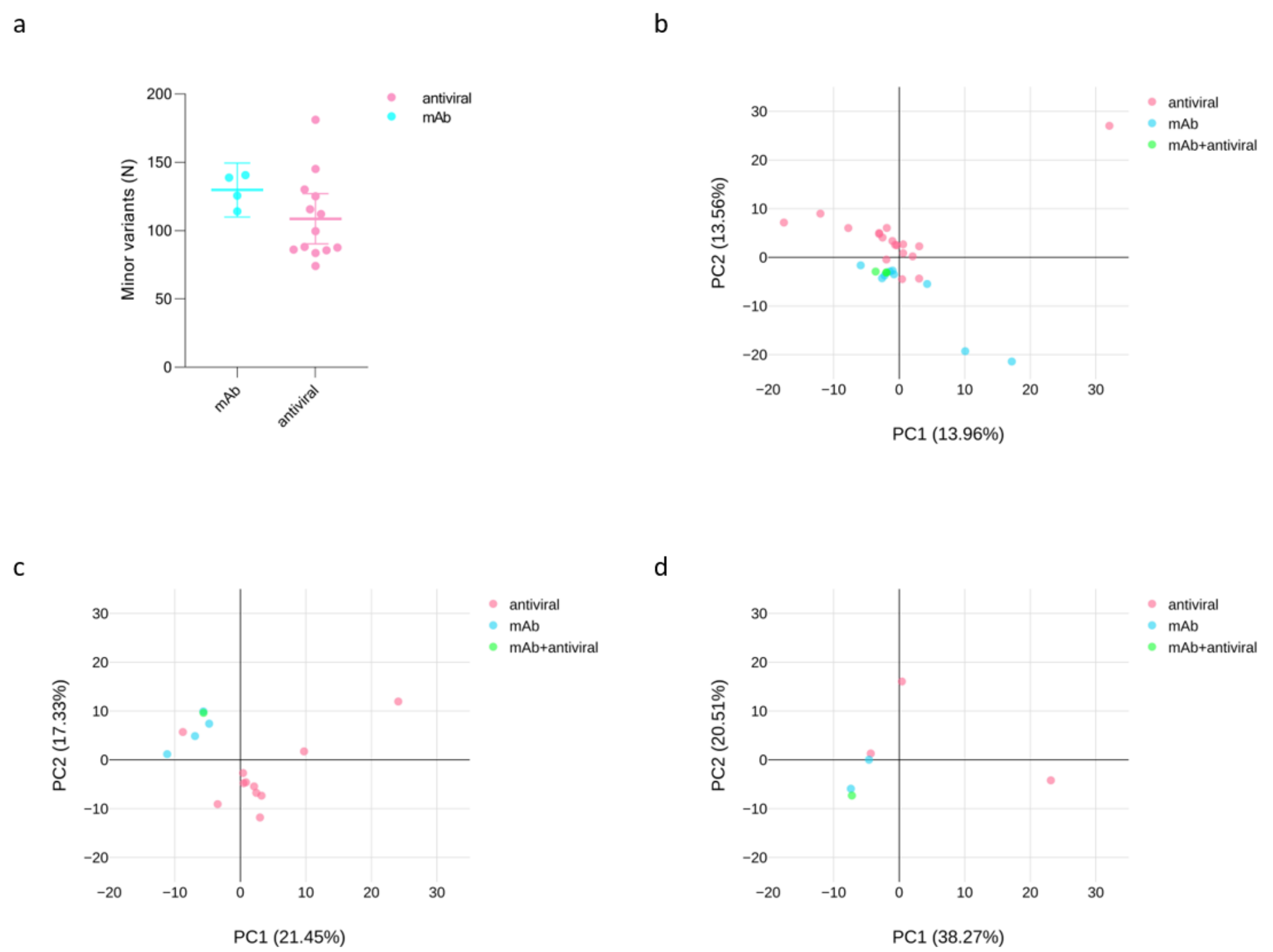

Figure S6. Treatments do not affect minor variants' number and type. *a) Comparison of the number of minor variants identified at any timepoint in subjects treated with monoclonal antibodies, depicted in light blue, treated with remdesivir, indicated in pink, and treated with a combination thereof, reported in green (N_{mAb}=4, $Mean_{mAb}$ =129.7, $N_{remdesivir}$=13, $Mean_{remdesivir}$=108.7, p=0.21, unpaired t test). b) Principal component analysis comparing the minor variants detected b) at any timepoint, c) at T0 d) and d) at T7 in subjects receiving remdesivir, monoclonal antibodies or a combination thereof. The variation explained by each PC is provided along the relative axis.*
